# Workplace tobacco cessation program in India: A success story

**DOI:** 10.4103/0019-5278.58919

**Published:** 2009-12

**Authors:** Gauravi A. Mishra, Parishi V. Majmudar, Subhadra D. Gupta, Pallavi S. Rane, Pallavi A. Uplap, Surendra S. Shastri

**Affiliations:** Department of Preventive Oncology, 3^rd^ Floor, Service Block, Tata Memorial Hospital, E. Borges Marg, Parel, Mumbai - 400 012, Maharashtra, India

**Keywords:** Contract employees, focus group discussions, tobacco cessation, urine cotinine, workplace

## Abstract

**Context:**

This paper describes the follow-up interventions and results of the work place tobacco cessation study.

**Aims:**

To assess the tobacco quit rates among employees, through self report history, and validate it with rapid urine cotinine test; compare post-intervention KAP regarding tobacco consumption with the pre-intervention responses and assess the tobacco consumption pattern among contract employees and provide assistance to encourage quitting.

**Settings and Design:**

This is a cohort study implemented in a chemical industry in rural Maharashtra, India.

**Materials and Methods:**

All employees (104) were interviewed and screened for oral neoplasia. Active intervention in the form of awareness lectures, focus group discussions and if needed, pharmacotherapy was offered. Medical staff from the industrial medical unit and from a local referral hospital was trained. Awareness programs were arranged for the family members and contract employees.

**Statistical Analysis Used:**

Non-parametric statistical techniques and kappa.

**Results:**

Forty eight per cent employees consumed tobacco. The tobacco quit rates increased with each follow-up intervention session and reached 40% at the end of one year. There was 96% agreement between self report tobacco history and results of rapid urine cotinine test. The post-intervention KAP showed considerable improvement over the pre-intervention KAP. 56% of contract employees used tobacco and 55% among them had oral pre-cancerous lesions.

**Conclusions:**

A positive atmosphere towards tobacco quitting and positive peer pressure assisting each other in tobacco cessation was remarkably noted on the entire industrial campus. A comprehensive model workplace tobacco cessation program has been established, which can be replicated elsewhere.

## INTRODUCTION

India is the second largest consumer of tobacco in the world. The prevalence of tobacco use among men has been reported to be high in most parts of the country (generally exceeding 50%). Its use is more common in rural areas as compared to urban areas.[1] Thirty per cent of the population 15 years or older (47% men and 14% of women) in India either smoked or chewed tobacco in 1998-99, which translates to almost 195 million people; 154 million men and 41 million women.[2] Hence, cessation should be a priority area for research in India. It has been estimated that US $1 investment on anti-tobacco activities would save US $13.[3] Research suggests that though 70% of tobacco users want to quit, only 3% are successful with will power alone.[4,5] Tobacco users are in dire need of support while quitting tobacco due to the addictive nature of tobacco products. Therefore, there is need of community based tobacco cessation facilities, which are less stigmatized like workplace and so more easily accessible.

Professional help is provided for tobacco users in tobacco cessation clinics to assist quitting. Though a variety of specialized services are made available at the clinic, the tobacco quit rates do not increase substantially, mainly because of poor follow-up of patients for counseling. With this background, the tobacco cessation programme was initiated at a chemical industry in Ratnagiri district of Maharashtra, India on World No Tobacco Day, 2007, considering its several advantages over a clinic based set up, mainly that of assured follow-up.

This program gave a unique opportunity to study the prevalence of tobacco use among the industrial employees and provide professional help to quit tobacco. Other objectives were to follow the tobacco users over a period of one year with active interventions and assess their tobacco quit rates and relapses, validate the self report tobacco history with rapid urine cotinine test, compare post-intervention knowledge, attitude and practices (KAP) regarding tobacco consumption with pre-intervention responses, assess the tobacco consumption pattern among the contract employees and provide them assistance to quit tobacco, reach the family members of employees with quit tobacco messages. The main aim, however, was to formulate a model work place tobacco cessation program which could be replicated in other work places to promote tobacco control activities. This paper enlists the follow-up interventions and over-all results.

## MATERIALS AND METHODS

This is an interventional cohort study of one year duration among 104 employees working in a chemical industrial unit at Ratnagiri, India. The detailed methodology and initial findings are explained in the previous paper.[6] The pre-intervention information on KAP regarding tobacco use was initially collected. This was followed by naked eyed oral examination of the employees to detect the oral precancerous lesions.[6] Thereafter, the tobacco users were followed up at an interval of six to eight weeks. The follow-up sessions comprised of focus group discussions (FGD) among tobacco users on various issues like motivation, changing attitude, coping with withdrawals, relapse prevention, sharing of experiences etc. During follow-up the employees were assisted with different types of relaxation and coping techniques, assertiveness skills and relapse prevention techniques. They were sensitized to the hazards of tobacco consumption, monitored for tobacco usage and assisted to cope with nicotine withdrawal symptoms. Numerous activities like slogan and poem competition, debates, feedback sessions and community group activities for tobacco control were conducted by the employees. Employees, in small groups conducted community activities to reach different sections of the society with ‘No Tobacco’ message. In addition to the follow-up visits, regular reminders in the form of phone calls and greeting cards with quit tobacco message, were sent to the tobacco users.

A lecture on different aspects of tobacco control was delivered by an expert during each visit for all employees. Follow-up oral examination was conducted for those diagnosed with lesions. Pharmacotherapy in the form of Bupropion tablets, an antidepressant which helps in reducing the cessation induced depression related to nicotine withdrawal, was prescribed from fifth session onwards, based on individual need assessment. Though many tobacco users are able to quit successfully with behavior therapy alone, pharmacotherapy, which focuses on alleviation of withdrawal symptoms, was expected to further increase the chance of successful quitting.[7] Nursing staff and doctors attached to the industry and from the local referral hospital were invited to participate as trainees during the active intervention sessions for local manpower development.

Post intervention data was collected at the end of 12 months from all employees. Urine was collected for conducting the rapid test for detection of cotinine in the urine thus validating the self reported tobacco history. This was done to add objectivity to the programme, so that, if successful, it can be replicated as a model to promote tobacco control activities at other workplaces.

### Principle and method of rapid urine cotinine test[8]

It is an immunoassay in which the chemically labeled cotinine (cotinine conjugate) competes with the free cotinine which may be present in the urine for limited antibody binding sites. In case of sufficient concentration of the drug, it fills the limited antibody binding sites. This prevents the attachment of the colored antibody-colloidal gold conjugate to the cotinine conjugate zone on the test band region. Therefore, absence of colored band on the test region indicates a positive result. The formation of a visible precipitant in the test zone occurs when the test urine is negative for the drug. The presence of a colored band in the control region serves as the verification that sufficient volume has been added, and that proper flow was obtained.

### Extension of the program

Since support of spouse and family members is important for the employee during the cessation period, an awareness lecture was conducted for family members of the selected industrial employees.

**Figure 1:**
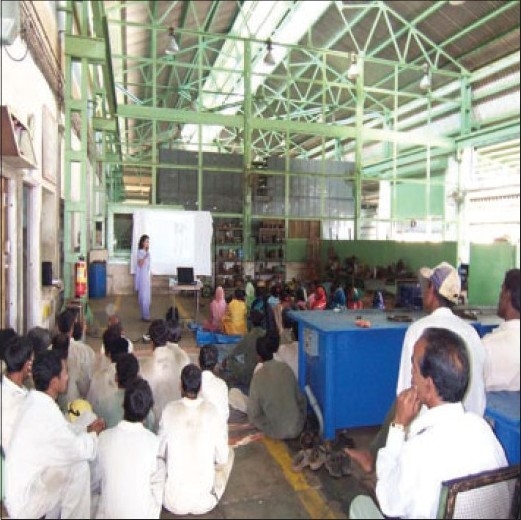
Awareness lectures for contract employees

Tobacco consumption was also common among the contract employees in the industry (initially they were not included in the program). As a demand from the employees and also taking advantage of the positive atmosphere created by the tobacco cessation program in the industrial campus, awareness lectures were conducted to cover the contract employees [Figure 1].

The data was computerized in FoxPro. Stata 8.2 was used to carry out statistical analyses. Change in pre-intervention and post-intervention KAP regarding tobacco use were analyzed on an intention-to-treat basis and the groups were compared using the usual non-parametric statistical techniques. Self reported tobacco cessation history was validated by rapid urinary cotinine test and the agreement rates were noted. In addition, results of the smoke check test[8] were used to validate cessation amongst smokers. The impact of the program was assessed by measuring the quit rates among tobacco users, as assessed by self-report history in addition to negative urine cotinine test results.

## RESULTS

The initial interview findings indicate tobacco consumption rate of 48% (50 tobacco users), of which seven employees used smoking forms, 33 smokeless forms and 10 used a combination of smoking and smokeless forms. According to the Fagerstorm scale, 13 employees (77%) using smoking form of tobacco had a score of zero, and 29 employees (67%) using smokeless forms of tobacco had a score varying from 0 to 5 [Table 1]. Smoke check test offered to smokers, was mainly used as an educational tool and the values of Carbon monoxide as detected by the smoke check test were 0-6 for 16 employees, greater than 10 for 1 employee.

**Table 1:** Severity of tobacco addiction and the quit rates

Employees using smoking forms of tobacco			Employees using non-smoking forms of tobacco		
	
Fagerstrom addiction score	No. of employees	Quit rates (%)	Fagerstrom addiction score	No. of employees	Quit rates (%)
0-2	17	03 (17.65)	0-2	05	02 (40)
3-5			3-5	24	12 (50)
6-8			6-8	12	05 (41.67)
9-10			9-10	01	
			11-13	01	
			14-16	00	

Tobacco users were offered behavioral therapy in the form of FGD and one-to-one counselling from round two onwards. Among the 50 tobacco users invited for the FGD, 90% participated in the first session, 88% in the second session, 88% in the third session, 66% in the fourth session, 90% in the fifth session, 84% in the sixth session and 86% in the seventh session. The main reason for non-participation was absenteeism on the days of intervention, which was high during fourth session due to festive season. The non-participants were contacted on phone to enquire about their stage of tobacco cessation and remind them about quitting tobacco. The tobacco cessation stage of tobacco users according to the follow-up sessions (by urine cotinine test during the final session and by self report history during all other sessions) are as indicated in [Table 2]. The number of employees quitting tobacco increased with each session of follow-up. The tobacco quit rates in the first, second, third, fourth, fifth and sixth follow-up sessions were 30%, 44%, 48%, 46%, 46% and 48% as per self report history. The quit rate at the end of the study was 40% as per the urine cotinine test results. Only five tobacco users were offered pharmacotherapy in the form of Bupropion from fifth session onwards, after careful assessment. A single employee among this group quit tobacco while two employees did not comply with the pharmacotherapy because of side effects like inability to concentrate and irritation following the use of Bupropion.

**Table 2:** Employee distribution according to stage of tobacco cessation in each follow-up (tobacco users: 50)

Stage of tobacco cessation	First follow up	Second follow up	Third follow up	Fourth follow up	Fifth follow up	Sixth follow up	Seventh follow up
Pre-contemplation	2	2	1	1	0	0	0
Contemplation	16	9	7	5	7	9	10
Preparation	17	17	18	20	18	16	19
Action	15	22	24	18	9	10	5
Maintenance	0	0	0	5	14	14	15
Relapse	0	0	0	1	2	1	1

One employee relapsed between third and fourth follow-up sessions and two employees relapsed between fourth and fifth follow-ups after initial quitting. In addition, one employee substituted tobacco chewing with tobacco application (masheri). One employee with relapse and one who had substituted with another form of tobacco later quit tobacco on re-counselling during the follow-up. The rapid urine cotinine test was performed among all employees at the end of one year of intervention. All employees who had reported as non users at the beginning of the study had a negative urinary cotinine test result. Among 24 employees who gave a self report history of tobacco cessation, 20 had negative urinary cotinine test results indicating they were true quitters while four had positive urine cotinine test results. All employees who self reported continuation of tobacco use tested positive by the rapid urinary cotinine test. The four employees with discrepancy in test results when taken into confidence and enquired again about tobacco usage, gave history of occasional tobacco use. The agreement rate between self reported tobacco history and the urine cotinine test results performed at the end of twelve months was 96% [Table 3].

**Table 3:** Agreement between self report tobacco history and rapid urine cotinine test at the end of 12 months of study

Self report tobacco history	Rapid urine cotinine rest results	
	
	Urine cotinine absent	Urine cotinine present
Tobacco non user or quit tobacco	71	04
Tobacco user	00	25

Kappa = 0.8987; *urine cotinine test was not done in 4 employees (1 tobacco user and 3 non users) as they were not available due to transfer or absenteeism

There was no difference in quit rates according to the age group of employees. The quit rates among employees using smoking forms of tobacco was 14.29%, those using tobacco in smokeless forms was 51.52% and 20% amongst those using combination of smoking and smokeless forms. Forty seven per cent of the employees who quit tobacco, had attempted quitting in the past. Among the 20 employees who finally quit tobacco, 21% had attempted quitting tobacco once previously, 10% had attempted quitting twice previously, 10% had attempted quitting thrice previously and one employee had attempted quitting seven times previously. Among the quitters, 53% had never attempted quitting in the past. They however quit tobacco after intense counselling by the tobacco control team. The tobacco quit rates among employees with presence of precancerous lesions was 25% and in employees without lesions was 30%. The oral examination findings correlate with the tobacco quit history and a remarkable reduction in the size of lesions and improvement in the oral hygiene was noted amongst employees who quit tobacco.

The correlation of tobacco quit rates on follow-up and the Fagerstorm scores, as assessed during the pre-intervention KAP, is as shown in Table 1. The tobacco quit rate among employees using smokeless tobacco with Fagerstorm score between 0-5 was 48.3%, as compared to 35.71% in employees with Fagerstorm score of more than 5.

**Figure 2:**
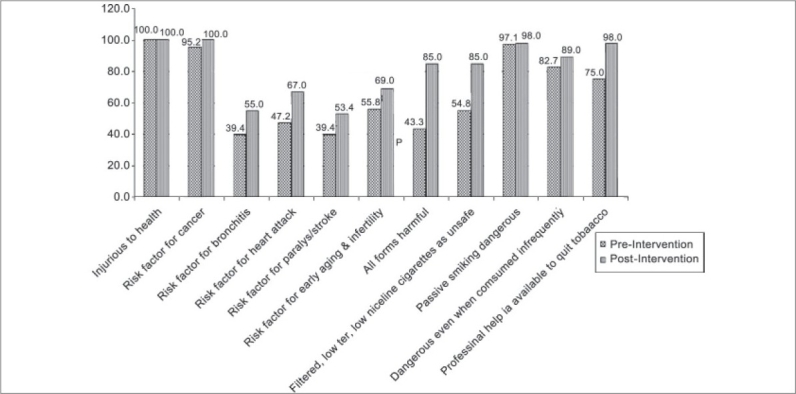
Comparison of pre-intervention and the post-intervention knowledge, attitude and practices among employees

The comparison of pre-intervention and the post-intervention responses among employees with respect to the knowledge regarding different aspects of tobacco is shown in [Figure 2]. There is considerable improvement in the knowledge regarding harms of tobacco in the post-intervention responses.

In the final session, employees gave feedback about the program. The employees found FGDs and health awareness lectures particularly useful. On being asked for suggestions for further improvement of the program, 54% of the participants appreciated the program and felt no improvement in the program was required. Some employees suggested introduction of periodic urinary cotinine test instead of a single test at the end, while few suggested that fear about acquiring tobacco related diseases needed to be introduced during counseling. The major withdrawal symptoms faced by the tobacco quitters were uneasiness, craving, temptation and constipation. When interviewed, lack of will power, to be part of a social group emerged as reasons given by non-quitters for continuation of tobacco use. Employees who relapsed after initial quitting stated physical discomfort like constipation and peer pressure from the social group outside the industry as the reasons for relapse. Majority of the employees appreciated the program and believed that it had helped them to bridge the gap between their thoughts and behavior and motivated them to stop tobacco use. This, they felt, was the major strength of the program.

**Figure 3:**
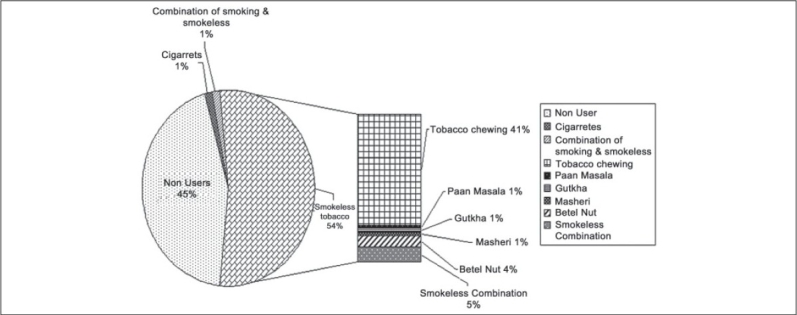
The different forms of tobacco usage among contract employees

**Figure 4:**
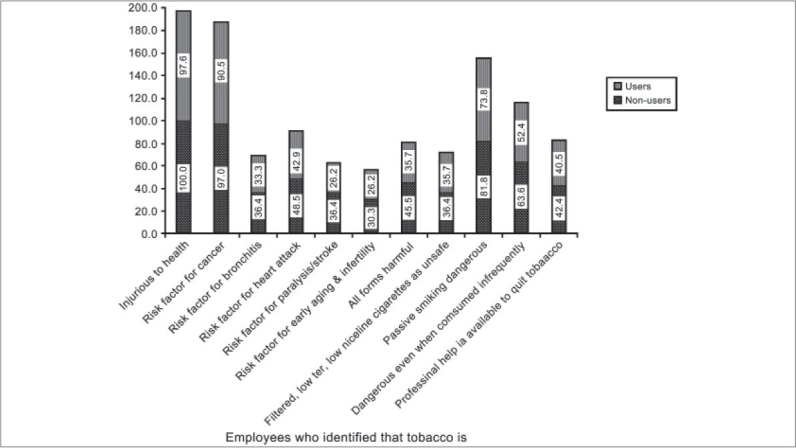
Pre-intervention knowledge, attitude and practices among the contract employees

On request from the participating industrial employees, two awareness sessions were conducted for the contract employees. There were 102 contract employees among which 100 participated in the health awareness sessions. Seventy five contract employees participated in the interviews among which 42 employees (56%) were tobacco users. The different forms of tobacco used by the contract employees are indicated in Figure 3. The socio-demographic profile of the contract employees is as shown in [Table 4]. This table shows that there is no difference between the tobacco users and non users with respect to any of the socio-demographic features. The pre-intervention KAP is as shown in Figure 4. After two sessions of awareness lectures 15 contract employees quit tobacco as reported by their supervisors. On oral examination 27 contract employees had the presence of oral precancerous lesions. An awareness session was also organized for family members of the employees and two amongst them quit tobacco.

**Table 4:** Socio-demographic characteristics of contract employees participating in the tobacco cessation program

Variables	Total participants (%)	Tobacco non users (%)	Tobacco users (%)	*P*-value
Total	75	33	42	
Age Groups (in years)				
≤30	33 (44.00)	17 (51.52)	16 (38.10)	*X*^2^ = 6.201
31-35	13 (17.33)	03 (9.09)	10 (23.81)	*P* = 0.287
36-40	17 (22.67)	09 (27.27)	08 (19.05)	
41-45	06 (8.00)	01 (3.03)	05 (11.90)	
46-50	03 (4.00)	02 (6.06)	01 (2.83)	
>50	03 (4.00)	01 (3.03)	02 (4.76)	
Mean age in years (SD)				
Education	32.72 (8.79)	31.15 (9.23)	33.95 (8.34)	
Primary [1-4]	07 (9.33)	01 (3.03)	06 (14.29)	*X*^2^ = 4.917
Secondary [5-10]	07 (9.33)	02 (6.06)	05 (11.90)	*P* = 0.296
Jr. College [11-12]	58 (77.33)	28 (84.85)	30 (71.43)	
Sr.College [13-15]	02 (02.67)	01 (3.03)	01 (02.38)	
Graduates and above	01 (1.33)	01 (3.03)	00	
Income per month				
Rs. 10,000-20,000	72 (96.00)	33 (100)	39 (92.86)	*X*^2^ = 2.455
Rs. 21,000-30,000	02 (2.67)	00	02 (4.76)	*P* = 0.293
Rs. >31,000	01 (1.33)	00	01 (2.38)	
Religion				
Hindu	64 (85.33)	29 (87.88)	35 (83.33)	*X*^2^ = 0.635
Muslim	04 (5.33)	01 (3.03)	03 (7.14)	*P* = 0.728
Others	07 (9.33)	03 (9.09)	04 (9.52)	
Duration of Service (in years)				
<5	47 (62.67)	22 (66.67)	25 (59.52)	*X*^2^ = 2.666
5-10	19 (25.33)	07 (21.21)	12 (28.57)	*P* = 0.615
11-15	02 (2.67)	00	02 (4.76)	
16-20	05 (6.67)	03 (9.09)	02 (4.76)	
>20	02 (2.67)	01 (3.03)	01 (2.38)	

## DISCUSSION

India is the second largest consumer of tobacco in the world, where tobacco is popular in smokeless forms as well. The available literature on tobacco cessation is mostly from the western countries and hence it was imperative to study it in the Indian context.

Tobacco use was mainly in smokeless forms, reflecting the cultural practices of the community. Very good participation rates were seen throughout the year, indicating that employees are interested in quitting tobacco and accept the program when conducted at their work site. The employees participated actively in the FGDs and discussed the difficulties encountered while quitting tobacco. Enormous support and motivation was provided by the tobacco quitters to help users to quit. Sharing of experiences and problem solving enhanced the quit rates. In a study in Austria, group counseling at the workplace was found to be an efficient method of smoking cessation.[9] In the present study, the employees were constantly reminded about quitting tobacco by phone calls and greeting cards sent with ‘quit tobacco’ message, in between the two follow-up rounds. At the end of the study, individual tobacco quitters were acknowledged by distributing pins stating “I am a proud tobacco quitter”. However, no monetary incentives were offered to the tobacco quitters. According to a smoking cessation program in California a multi-component program with telephone counseling may be as effective as a program plus incentives and competition for long-term smoking cessation and more effective than a traditional program.[10]

The tobacco quit rate in the pharmacotherapy group was 20%. Three employees relapsed and one employee substituted with other forms of tobacco while trying to quit. Immediate support could be provided to them as this was an on-going and sustainable activity. Tobacco is highly addictive and hence the participants need continuous support during the quitting phase to prevent relapse and substitution and sustain quitting. In one of the meta-analysis on workplace smoking cessation interventions initial effectiveness was seen, however the effect decreased over time and was not present beyond 12 months.[11] In a tobacco cessation program in a workplace in the UK using behavioral support and nicotine patches, 20% quit smoking at end of 12 months. Five per cent of employees relapsed and after trying again quit successfully.[12] Hence, to maintain the employees in the cessation phase and prevent relapse, after the program completion, tobacco cessation information in the form of pamphlets, booklets, videotapes has been made available by this study at the worksite. The medical team from the industry and local hospital were thoroughly trained to offer tobacco cessation services in order to ensure continuity of support. A list of tobacco cessation referral resources and addresses was provided to the employees.

Studies have revealed that simple advice from the physician increases abstinence rates significantly (by 30%) than no advice.[13] Likewise, nursing led interventions for smoking cessation increased the chances of successfully quitting by 50%.[14] A brief face to face intervention at work site by occupational physician during the mandatory annual examination gave 36% higher cessation rate than a simple advice intervention.[15]

Urine cotinine test done at the end of the study had an agreement rate of 96% with the self reported tobacco history. Self-reported smoking status among participants in a lung cancer screening trial was highly consistent with urinary cotinine test results.[16] 40% of tobacco users in the present study quit tobacco at the end of one year. The quit rate was higher among employees using smokeless forms as compared smoking forms and was not related to the age group of employees. 47% of the employees who quit tobacco had made attempts to quit tobacco in the past.

The prevalence of oral precancerous lesions was higher among the users of smokeless tobacco. Majority of the lesions decreased in employees who quit tobacco. Contrary to our expectations tobacco quit rates are much lower among employees with presence of oral pre-cancerous lesions as compared to those without lesions. Higher frequency and duration of tobacco use, which was noticed among the employees with presence of lesions, may have been responsible for this. This was also indicated by employees with oral pre-cancerous lesions presenting with higher Fagerstorm scores. Higher tobacco quit rates were noted in employees with lower Fagerstorm score of smokeless tobacco. The employees were interviewed at the end of the study on various aspects of tobacco use. A considerable improvement in the knowledge regarding harms of tobacco was seen in the post-intervention period.

In our study, the employees found FGDs and health awareness lectures particularly useful in quitting tobacco. Regular follow ups, persistence, good communication skills and commitment by the team of program providers were appreciated by all. In a workplace tobacco cessation program in the UK, 52% of industrial employees stated regular face to face contact and monitoring of the progress particularly useful in stopping smoking.[12]

The tobacco consumption rate was 56% among the contract employees, mainly smokeless forms. Majority of the contract employees were illiterates. Health awareness using audio-visual aids was conducted, which received active participation and a tobacco quit rate of 35.71% was reported by their supervisors. The prevalence of oral precancerous lesion was very high (55.1% of tobacco users) among the contract employees and it correlates with their tobacco consumption habit. There was no significant difference in the socio-demographic characteristics between the tobacco users and the non users.

Family members play an important role in supporting the tobacco user during the quitting phase. It is also important that the family members understand the harms of tobacco and quit the habit themselves. Hence awareness sessions were arranged for the family members in the local community. These sessions had good participation and some of them quit tobacco. This project not only worked intensely with the individual tobacco user but also went ahead to bring about a change in the social environment by educating the contract employees and family members of the permanent employees. The employees participated in societal initiatives to spread awareness regarding harms of tobacco in the local community. It is necessary to promote change in social norms by de-moralizing tobacco use through awareness about its harms as it encourages smokers in their attempts to quit.[7]

This program brought about a few admirable results -it helped develop a positive atmosphere towards tobacco quitting, remarkably noted on the entire industrial campus. The positive peer pressure assisted each other in tobacco cessation. As the occupational health personnel in the industrial unit were involved at every stage of the program and trained in tobacco cessation, they acted as continuous support to the employees who approached them regarding any difficulties encountered during cessation. This health team of the industry is expected to provide independent professional support for tobacco cessation activities in future. Overall, a committed team, working for tobacco cessation and a motivated management and union cooperating with program providers, contributed to the great success of the program.

The program changed the atmosphere regarding tobacco consumption on the entire industrial campus, thus exemplifying how peer pressure can bring about a positive change in the life style of entire group with spill over to the community. The program has not only created a healthy workplace at the selected industrial unit but has also served as an excellent opportunity to impart training on tobacco cessation to the health care professionals in the industry and at the local referral hospital for capacity building in tobacco cessation activities. It has succeeded in reaching out to the local community with the ‘quit tobacco’ message. A model workplace tobacco cessation and oral cancer screening program has been established at the industrial unit, which can be used as a model to promote tobacco control activities at other workplaces.
